# Early alcohol abstinence symptoms and the role of cumulative adversity

**DOI:** 10.1111/acer.70137

**Published:** 2025-09-29

**Authors:** Helen C. Fox, Jorge Alcina, Scott M. Hyman, Verica Milivojevic, Rajita Sinha

**Affiliations:** ^1^ Department of Psychiatry, Yale School of Medicine Yale Stress Center New Haven Connecticut USA; ^2^ Department of Psychiatry, Indiana School of Medicine Indiana University Indianapolis Indiana USA; ^3^ Clinical Psychology Program, Miami Campus Albizu University Miami Florida USA

**Keywords:** alcohol craving, cardiovascular, cumulative adversity, early abstinence, mood

## Abstract

**Background:**

This study examined the course of early alcohol abstinence symptoms across multiple clinical domains (i.e., cravings, withdrawal, mood, and cardiovascular measures) in individuals undergoing inpatient alcohol treatment and assessed whether cumulative lifetime adversity influences the severity and trajectory of these symptoms.

**Methods:**

Researchers tracked withdrawal symptoms, alcohol cravings, mood states, heart rate, and blood pressure in 34 inpatient participants at treatment admission and weekly for three to four consecutive weeks. The analysis employed two approaches: first, examining symptom presentation and progression over time in alcohol‐dependent individuals using cumulative adversity as a moderating variable; second, comparing symptom patterns between alcohol‐dependent participants with high versus low lifetime adversity against 38 control participants at each timepoint.

**Results:**

Abstinence symptoms resolved by the third week of inpatient treatment across all participants. However, alcohol‐dependent individuals with greater lifetime adversity exhibited significantly more severe symptom patterns compared to alcohol‐dependent individuals with fewer adverse experiences. These differences persisted even when controlling for recent alcohol and tobacco use severity over the preceding 3 months.

**Conclusions:**

Understanding the profile and progression of early abstinence symptoms, along with stress‐related moderating factors, could inform more personalized care planning. Cumulative lifetime adversity may serve as a readily measurable correlate of early abstinence severity and may be valuable for predicting alcohol treatment outcomes. Addressing the effects of cumulative lifetime adversity may serve as a target for early intervention in patients with alcohol use disorder.

## Graphical abstract

Objectives were to characterize early alcohol abstinence symptoms during inpatient treatment, and examine the role of cumulative lifetime adversity in moderating these symptoms. A decrease in alcohol craving, anxiety, and depression was observed in all participants by week 3 of treatment. A more severe abstinence profile was seen in individuals with higher adversity, particularly on admission to treatment. As elevations in these clinical symptoms on treatment entry can predict treatment success, cumulative adversity may represent a key indicator of risk.

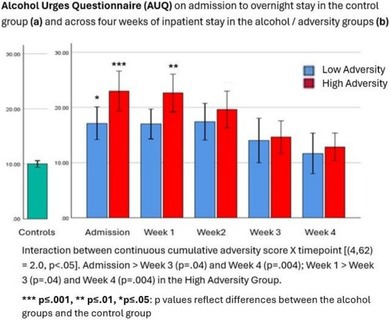

## INTRODUCTION

Cumulative adversity refers to the buildup of negative life events across an individual's lifespan. These can range in severity from challenging life circumstances such as financial, health, and relationship issues to life‐threatening traumatic incidents such as sexual and physical violence (Turner & Lloyd, 1995). Notably, as experienced life events, cumulative adversity can be considered independent of subjective perceived stress, as it represents an objective index of chronic “wear and tear” across multiple interconnected biobehavioral systems (Gustafsson et al., 2012; Seo et al., 2014), known as “allostatic load” (McEwen & Stellar, 1993). In support of this, cumulative adversity has been associated with a range of neurocognitive (Ansell, Rando, et al., 2012; D'Amico et al., 2022; Seo et al., 2014), physiological, metabolic, and inflammatory (Dich et al., 2015; Finlay et al., 2023; Lampert et al., 2016; Li et al., 2023) adaptations in children, adolescents, and adults, contributing to the etiology and severity of many chronic stress‐related disorders (Guidi et al., 2021) including substance use disorders (SUD) (Koob & Colrain, 2020; Koob & Schulkin, 2019).

For example, approximately 70% of individuals with alcohol use disorder have endured at least one form of childhood trauma (Schwandt et al., 2013), and lifetime cumulative exposure to both distal and proximal adversity (as defined by lifetime exposure to major and potentially traumatic experiences) has also been linked to substance use disorders in young adults (Turner & Lloyd, 2003). Additionally, a review by Lijffijt et al. (2014) concluded that both childhood and cumulative stressful life events are associated with increased risk of alcohol use disorders across all stages of initiation, regular, and pathological use. Importantly, as stress pathophysiology has been shown to underpin craving and relapse in substance and alcohol use disorder (Fox et al., 2007; Sinha, Fox, et al., 2011a; Syed et al., 2023) it is possible that cumulative adversity might represent a key indicator of core clinical features during addiction milestones such as the period of early abstinence.

While acute alcohol withdrawal refers to a syndrome with well‐defined physiological signs or symptoms (e.g., tremors, agitation, night sweats, delirium tremens) ranging from mildly uncomfortable to life‐threatening, and beginning hours after cessation and lasting a few days (American Psychiatric Association, 2022), subsequent early abstinence symptoms reflect a milder intermediate stage absent of these specific physiological symptoms that characterize alcohol withdrawal *syndrome* (Heilig et al., 2010; Sinha, Shaham, & Heilig, 2011). Rather, early abstinence is characterized by negative mood, anxiety and craving sensitization, anhedonia, irritability, sleep disturbances, cardiovascular changes, and emotional lability, where disturbances in stress responding are most pronounced (Fox et al., 2007; Schwarze et al., 2024; Sinha, Fox, et al., 2011). Despite these symptoms representing key prognostic indicators of clinical outcome and intervention (Martins et al., 2022), potentially to a greater extent than acute withdrawal syndrome (Heilig et al., 2010), they are not typically systematically monitored in treatment settings (Martins et al., 2022; Sinha et al., 2021). Hence, limited evidence‐based research exists regarding the effects of abstinence initiation on craving, negative mood, and postacute withdrawal symptoms (including cardiovascular output) (Hallgren et al., 2018). In addition, as the high prevalence of cumulative lifetime adversity in alcohol use disorders suggests a reciprocal relationship subserved by overlapping stress system pathophysiology, the cumulative impact of adverse events across the lifespan may moderate early withdrawal symptoms.

In view of this, objectives are first, to systematically characterize the clinical presentation of early abstinence symptoms from alcohol across three to 4 weeks of inpatient treatment. To date, these symptoms are not well characterized, and they may highlight important indicators of treatment success. Second, we examine whether the nature and severity of early abstinence symptoms are moderated by cumulative life adversity on dimensions of craving, mood, anxiety, and general withdrawal symptoms, including cardiovascular output. Findings will help establish whether cumulative adversity can represent a useful objective index for identifying patients who may need more intensive support during early recovery as well as help select appropriate treatment interventions based on individual risk profiles.

## METHODS

We conducted a secondary analysis from data collected between January 2012 and July 2016, as part of a laboratory study focused on immune response to stress in individuals with high and low depressive symptomology and who met DSM‐IV‐TR criteria for alcohol dependence (Fox et al., 2020). All participants provided written and verbal consent, and the Human Investigation Committee of the Yale University School of Medicine approved the study.

### Participants

Thirty‐four treatment‐seeking individuals with alcohol dependence and 38 light social drinkers were recruited via flyers and advertisements placed in and around the New Haven, Connecticut area. Alcohol‐dependent participants were admitted for 3 to 4 weeks of inpatient treatment, and exclusion criteria included DSM‐IV‐TR dependence for any drug other than alcohol or nicotine and any current psychiatric illness requiring medication, apart from stabilization on SSRIs. All participants underwent electrocardiography and laboratory tests of renal, hepatic, pancreatic, hematopoietic, and thyroid function to ensure good physical health. All controls were light social drinkers (25 drinks or less per month) as classified by the Cahalan Quantity‐Frequency Variability Index (Cahalan et al., 1969), and were excluded if they met current or lifetime dependence criteria for alcohol or any other illicit substance.

### General procedures

Alcohol‐dependent participants were admitted to the Clinical Neuroscience Research Unit (CNRU) of the Connecticut Mental Health Center (CMHC) for 3 to 4 weeks of standard individual and group counseling treatment for alcohol dependence (Mercer & Woody, 2005). The CNRU is a locked inpatient treatment research facility, with no access to alcohol or other substances, and limited access to visitors. Upon admission, participants completed structured assessments measuring psychiatric and substance use history as well as a subjective measure of cumulative adversity. They also completed measures of alcohol withdrawal, mood, and craving upon admission and on a weekly basis until treatment discharge. All participants completed three full weeks of assessments; however, only a third of the alcohol sample (*N* = 12) remained in treatment long enough to complete week 4 assessments. Control participants were admitted to the Hospital Research Unit (HRU) of the Yale Clinical Center of Investigation (YCCI), located at Yale/New Haven Hospital (YNHH) for a 3‐night stay in a similar environment, as part of the primary study (Fox et al., 2020). They completed identical assessments on the initial day of stay, which were compared with the weekly data from the alcohol‐dependent inpatients.

### Assessments

#### Lifetime cumulative adversity

To examine the role of cumulative lifetime adversity on early abstinence, we used the cumulative adversity interview (CAI), adapted from Turner and Lloyd (2003), which is a semi‐structured interview administered by trained staff. The 140‐item interview is a comprehensive measure of cumulative adversity that covers recent and major life events, life trauma, and chronic stressors, and has demonstrated predictive validity in a range of health‐related conditions (Künzi et al., 2022; Slavich et al., 2019).

The scale *recent life events*: comprises a checklist of 33 items referring to discrete stressful events occurring in the previous 12 months. These are broadly divided into items referring to exits from the social field (e.g., death, divorce, relationships ending) and undesirable events, both interpersonal and financial (e.g., being attacked, financial crises, robberies). *Major life events*: this includes 11 items relating to social adversities, not typically violent in nature, but differing from standard life events due to severity and potentially long‐term consequences. Examples include parental divorce and failing a grade in school. *Life traumas*: This section comprises 34 items relating to life trauma, witnessed violence, and traumatic news. This includes events that imply force or coercion and include physical, emotional, and/or sexual abuse, including rape and being injured with a weapon. Witnessed violence contains items including being present in dangerous or upsetting situations, and traumatic news relates to hearing about someone else being killed, abused, or injured. *Chronic life events*: This section comprises 62 items relating to subjective responses to continuous stressors or ongoing life problems including interpersonal, social, and financial difficulties. While the chronic life events scale represents a subjective response to ongoing adversity, the other three categories pertain to a discrete number of objective adverse events. As such, in the present paper, we combined the cumulative adverse life events (CALE) count from the three objective subscales (Major Life Events, Life Trauma, Recent Life Events) to provide a continuous score of cumulative lifetime adversity (Ansell, Gu, et al., 2012; Stults‐Kolehmainen et al., 2014). In all cases, a higher score pertained to a greater number of adverse events.

#### Subjective weekly assessments

The following questionnaires were administered upon inpatient admission, and then subsequently once per week for the following 3 to 4 weeks.

##### The Revised Clinical Institute Withdrawal Assessment for Alcohol (CIWA‐Ar) (Sullivan et al., 1989)

The CIWA is a 14‐item structured questionnaire based on semi‐quantitative evaluation, used to systematically assess the severity of alcohol withdrawal symptoms. Pulse and blood pressure are assessed as part of the CIWA‐Ar, and withdrawal severity is rated along 10 different dimensions including: agitation, anxiety, sensory clouding, headache, nausea or vomiting, paroxysmal sweats, tremors, and auditory, tactile, or visual disturbances. Ratings are both observational and subjective, with scores in each category ranging in severity from 0 to 7. An overall score of <8 to 10 suggests mild withdrawal symptoms, a score of 8 to 15 suggests moderate symptoms, and a score of 15 or more suggests severe withdrawal, with a probable medically assisted detoxification required. This is a widely used measure and has well documented reliability, reproducibility, and validity, based on high correlations with ratings by expert clinicians (Wiehl et al., 1994).

##### The Profile of Mood States (POMS) (Lorr et al., 2003)

The POMS comprises a list of 72 mood‐related words (e.g., happy, angry, tense, shaky) that are rated on a scale from 0 (not at all) to 4 (extremely) according to the extent to which a participant feels each emotion at a point in time. All 72 items are collapsed into six mood dimensions (Anxiety‐Tension, Anger‐Hostility, Depression‐Dejection, Vigor‐Activity, Fatigue‐Inertia, Confusion‐Bewilderment). The scale has demonstrated high test re‐test validity, construct validity, and good internal consistency (0.78–0.90) (O'Halloran et al., 2004).

##### The Beck Depression Inventory (BDI) (Beck et al., 1961)

The BDI is a sensitive 21‐item self‐report measure of depression symptoms. Items are anchored from 0 (low depression) to 3 (high depression) to quantify the intensity of each symptom. A total score of 10–18 typically indicates mild–moderate depression; 19–29, moderate–severe depression; and 30–63, severe depression. The scale is extensively used in experimental and clinical settings and has shown high content, concurrent, and construct validity.

##### The Alcohol Urge Questionnaire (AUQ) (Bohn et al., 1995)

The AUQ is a valid and reliable eight‐item state measure that assesses participants' urge for an alcoholic drink at the time the questionnaire is completed. All eight items are in the form of craving‐related statements, and item scores range from 1 (strongly disagree) to 7 (strongly agree). The instrument has been found to be significantly associated with drinking measures and dependence severity (Fox et al., 2007; MacKillop, 2006).

### Statistical analysis

All statistical analyses were conducted using SPSS for Windows version 26. Demographic and substance use variables were analyzed using chi‐square tests and ANOVAs to identify group differences (Table 1). Two primary analyses were performed for each outcome measure:

- *Linear mixed effects (LME) models* examined changes in abstinence symptoms over the 3 to 4‐week inpatient treatment period, analyzing data from alcohol‐dependent individuals. The models incorporated total Cumulative Adverse Lifetime Events (CALE) count scores as a between‐subjects factor and Week as a within‐subjects factor (Baseline, Week 1, Week 2, Week 3, Week 4). All CALE scores underwent mean‐centering prior to analysis and were treated as continuous variables. Individual subjects served as the random effects component, and a compound symmetry covariance structure was implemented across all models. In all LME analyses, total alcohol consumption (number of drinks) and tobacco use (number of cigarettes) over the preceding 3 months were included as covariates. The LME approach was selected for its advantages in repeated measures designs, notably its ability to retain participants with incomplete data, rather than excluding cases with missing observations (Gabrio et al., 2022). When significant interactions involving CALE scores emerged, posthoc analyses were conducted using a median split approach. This technique divided participants into high and low cumulative lifetime adversity groups using a median split based on their continuous scores, facilitating simple effects analyses and enabling clearer graphical representation of the interaction patterns.
- *One‐way analyses of covariance* (ANCOVAs) were conducted to evaluate differences between controls (measured at baseline only) and both low adversity and high adversity alcohol‐dependent groups at each timepoint separately. A median split of the CALE continuous score was used to delineate the adversity groups. The CALE variable's normal distribution (Kolmogorov–Smirnov test: *p* = 0.18) supports the use of median splits, which are most effective with symmetrical data (DeCoster et al., 2011). In all ANCOVA analyses, sex, total alcohol use, and total tobacco use over the preceding 3 months were included as covariates.

**TABLE 1:** Demographics & alcohol use.

	Controls (C) (*n* = 38)	Alcohol low (L) adversity (*n* = 17)	Alcohol high (H) adversity (*n* = 17)	*p*	Simple effects
Gender (% male)	19 (50%)	15 (88%)	12 (70%)	0.02	(C) vs. (L, H)
Age	33.5 ± 10.4	39.7 ± 9.1	39.1 ± 9.9	ns	
Years of education	15 ± 2.7	13.6 ± 1.7	12.6 ± 1.6	0.002	(C) vs. (L, H)
Race/Ethnicity				ns	
Caucasian	16 (42%)	9 (53%)	5 (30%)		
African American	15 (40%)	7 (41%)	12 (70%)		
Hispanic	3 (8%)	1 (6%)	0 (0%)		
Asian	2 (5%)	0 (0%)	0 (0%)		
Other	2 (5%)	0 (0%)	0 (0%)		
Cumulative adverse life events (CALE)					
Total scores for objective CALE scales	9.6 ± 6.1	9.5 ± 3.3	24.7 ± 7.7	<0.0001	(C, L) vs. (H)
Major life events	2.0 ± 1.8	2.1 ± 1.3	5.1 ± 2.1	<0.0001	(C, L) vs. (H)
Recent life events	2.4 ± 2.3	1.4 ± 1.2	4.5 ± 3.8	0.002	(C, L) vs. (H)
Life trauma	5.3 ± 3.9	6.1 ± 2.8	15.2 ± 5.0	<0.0001	(C, L) vs. (H)
Alcohol use					
Days abstinent in past 3 months	72.9 ± 10.9	44.9 ± 23.5	26.4 ± 24.6	<0.0001	(C) vs. (L) vs. (H)
Days of use in past 3 months	17.1 ± 10.9	45.1 ± 23.5	63.6 ± 24.6	<0.0001	(C) vs. (L) vs. (H)
Mean no. of drinks per use	2.4 ± 3.5	9.4 ± 6.1	12.4 ± 11.1	<0.0001	(C) vs. (L, H)
Total no. of drinks in past 3 months	41.1 ± 30.1	477.3 ± 529.9	917 ± 1034.7	<0.0001	(C) vs. (L) vs. (H)
CIWA scores on admission	0.4 ± 0.6	2.5 ± 3.1	4.6 ± 4.0	<0.001	(C) vs. (H)
Cigarette use					
Days abstinent in past 3 months	78.7 ± 28.3	59.6 ± 38.7	16.4 ± 35.0	<0.0001	(C, L) vs. (H)
Days of use in past 3 months	11.3 ± 28.3	30.4 ± 38.7	73.7 ± 35.0	<0.0001	(C, L) vs. (H)
Mean no. of cigarettes per day	4.2 ± 3.5	7.1 ± 4.4	7.3 ± 6.3	<0.0001	ns
Total no. of cigarettes in past 3 months	203.2 ± 339	485 ± 472.8	617.5 ± 328	0.01	(C, L) vs. (L, H)
Clinical profile					
Current mood disorder – no. of patients	1 (3%)	0 (0%)	4 (24%)		
Lifetime mood disorder – no. of patients	0 (0%)	2 (12%)	6 (35%)		
Current anxiety disorder – no. of patients	1 (3%)	0 (0%)	1 (6%)		
Lifetime anxiety disorder – no. of patients	2 (5%)	1 (6%)	5 (29%)		

All analyses were Bonferroni corrected to minimize the likelihood of Type 1 errors. Years of education were excluded from the covariate set after preliminary analyses revealed no significant associations between educational attainment and any of the dependent variables under investigation.

## RESULTS

### Participants

The control group had significantly more years of completed education than both the low and high adversity alcohol‐dependent groups and comprised more women than the two alcohol‐dependent groups (Table 1). In addition, the high adversity alcohol‐dependent group reported greater severity of both alcohol and tobacco use compared with the low adversity alcohol‐dependent group and controls.

All results from the LME and univariate models presented in the text include the addition of covariates.

### Alcohol craving (alcohol urges questionnaire – AUQ)

#### Analysis 1: Effects of cumulative lifetime adversity on alcohol craving at all weekly timepoints in individuals with alcohol dependence

**FIGURE 1:**
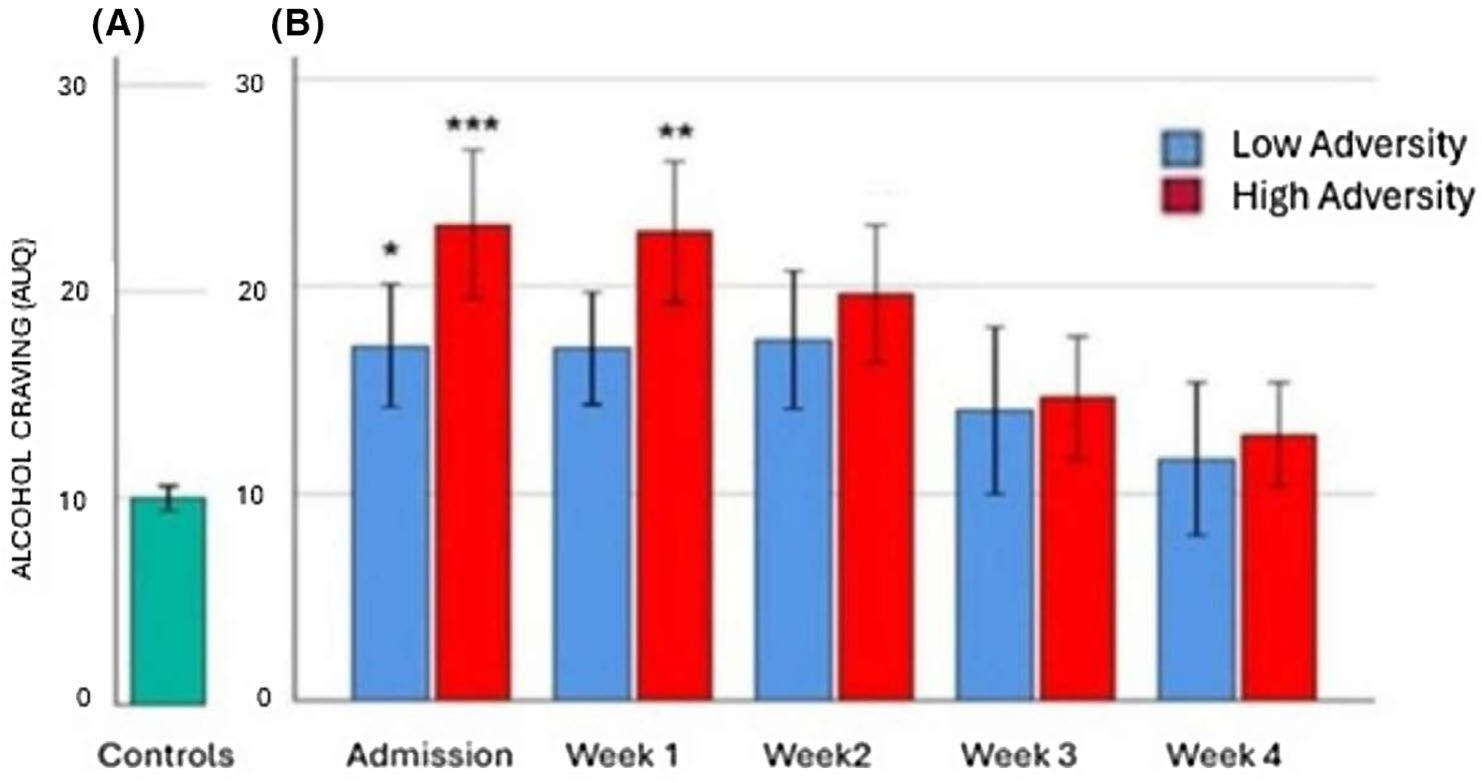
Alcohol craving. Alcohol Urges Questionnaire (AUD) on admission to overnight stay in the control group (a) and across 4 weeks of inpatient stay in alcohol/adversity groups (b). Interaction between continuous cumulative adversity scores × timepoint [*F* (4, 62) = 2.0, *p* < 0.05]. In the high adversity group: Admission >week 3 (*p* = 0.04) and week 4 (*p* = 0.004) and week 1 >week 3 (*p* < 0.04) and week 4 (*p* = 0.004). ***p ≤ 0.001, **p ≤ 0.01, *p ≤ 0.05. All *p* values represent the difference between the alcohol groups and the control group.

A main effect of Timepoint [*F* (4, 62) = 7.7, *p* < 0.001] indicated a decrease in alcohol craving at week 3 compared to admission (trend, *p* = 0.07) and a significant decrease in alcohol craving at week 3 of inpatient treatment compared to week 2 (*p* = 0.05) (Figure 1). A significant interaction was also observed between Cumulative Adverse Lifetime Events (CALE continuous scores) and weekly timepoints [*F* (4, 62) = 2.0, *p* < 0.05]. Posthoc analysis using High Adversity and Low Adversity alcohol groups indicated that alcohol craving was significantly higher on admission and week 1 compared with weeks 3 and 4 of inpatient stay (admission > week 3, *p* = 0.04 and week 4, *p* = 0.004. Week 1 > week 3, *p* = 0.04 and week 4, *p* = 0.004) in the High Adversity alcohol group only. No differences in alcohol craving were observed between the timepoints in the Low Adversity alcohol group.

#### Analysis 2: Effects of group (Controls vs. Low Adversity alcohol group vs. High Adversity alcohol group) on craving at each weekly timepoint

A main effect of Group was observed on admission [*F* (2, 66) = 7.6, *p* = 0.001], where both the High (*p* = 0.001) and Low (*p* = 0.04) Adversity groups reported significantly higher alcohol craving compared with controls. A main effect at Week 1 [*F* (2, 64) = 5.5, *p* = 0.007] also indicated that the High Adversity group reported significantly elevated alcohol craving compared with controls (*p* = 0.006; Figure 1).

### Depressive symptoms (Beck Depression Inventory – BDI)

#### Analysis 1: Effects of cumulative lifetime adversity on depressive symptoms at all weekly timepoints in individuals with alcohol dependence

**FIGURE 2:**
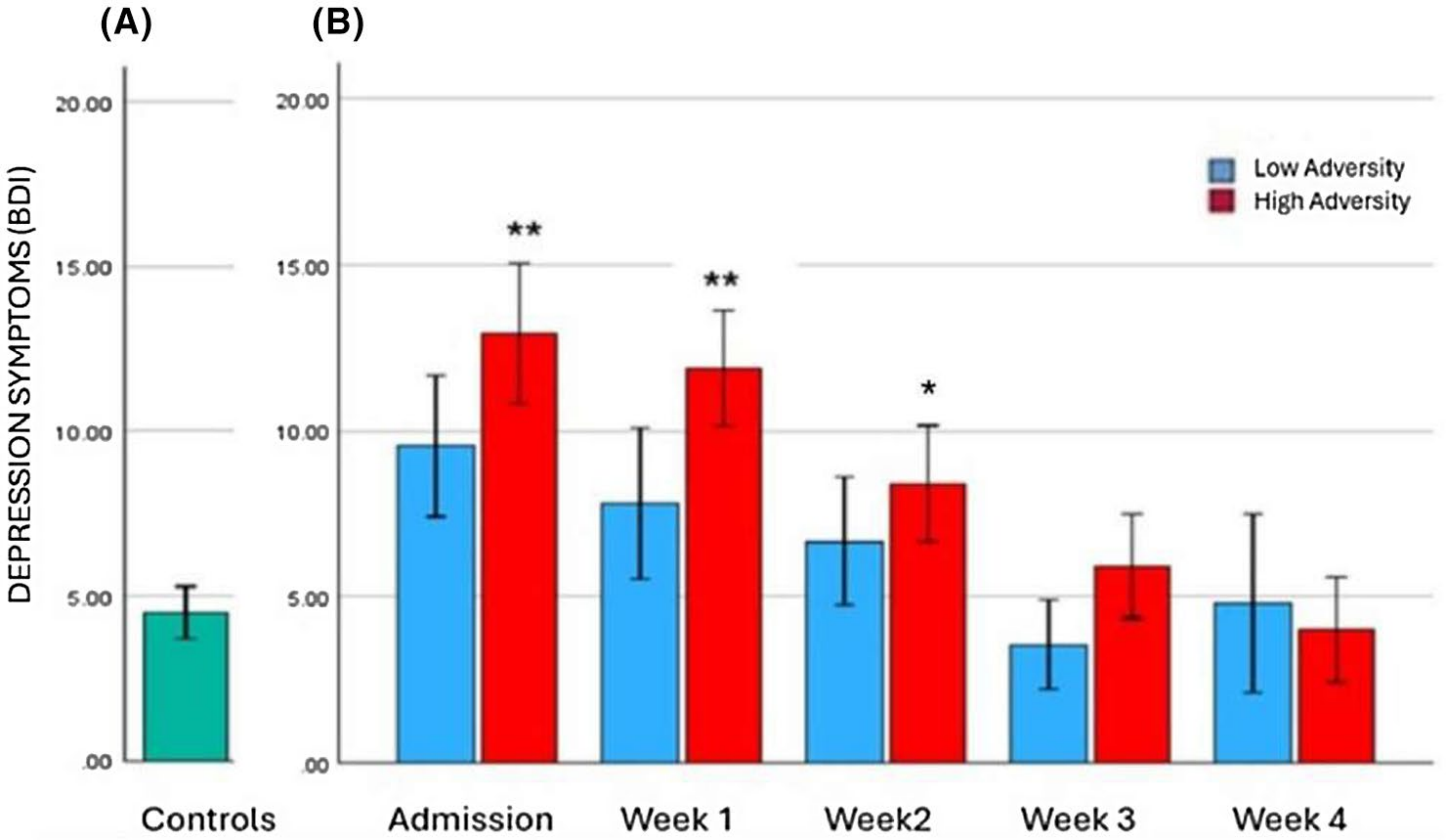
Depression. Beck Depression Inventory (BDI) on admission to overnight stay in the control group (a) and across 4 weeks of inpatient stay in alcohol/adversity groups (b). A main effect of timepoint [*F* (4, 65) = 7.9, *p* < 0.001]. Admission >week 3 (*p* = 0.02). ***p ≤ 0.001, **p ≤ 0.01, *p ≤ 0.05. All *p* values represent the difference between the alcohol groups and the control group.

A main effect of Timepoint [*F* (4, 65) = 7.9, *p* < 0.001] indicated higher depressive symptoms reported at baseline compared with Week 3 (*p* = .02) in the alcohol‐dependent participants (Figure 2).

#### Analysis 2: Effects of Group (Controls vs. Low Adversity alcohol group vs. High Adversity alcohol group) on depressive symptoms at each weekly timepoint

A main effect of Group on admission [*F* (2, 64) = 10.5, *p* < 0.001] indicated that the High Adversity alcohol group reported greater depressive symptoms compared with both the control (*p* < 0.001) and the Low Adversity alcohol (*p* < 0.03) groups. The Low Adversity alcohol group also reported higher levels of depressive symptoms compared with the controls, but this was a trend only (*p* <. 06). At Week 1, a main effect of Group [*F* (2, 77) = 10.8, *p* < 0.001] showed that the High Adversity alcohol group reported higher depressive symptoms compared with both the control (*p* < 0.001) and the Low Adversity (*p* = 0.004) groups. At Week 2, a significant main effect of Group [*F* (2, 64) = 37, *p* = 0.03] indicated that the High Adversity group reported significantly greater ratings of depressive symptoms compared to the control group (*p* < 0.03).

### Anxiety‐tension (POMS)

#### Analysis 1: Effects of cumulative lifetime adversity on anxiety‐tension dimensional symptoms at all weekly timepoints in individuals with alcohol dependence

**FIGURE 3:**
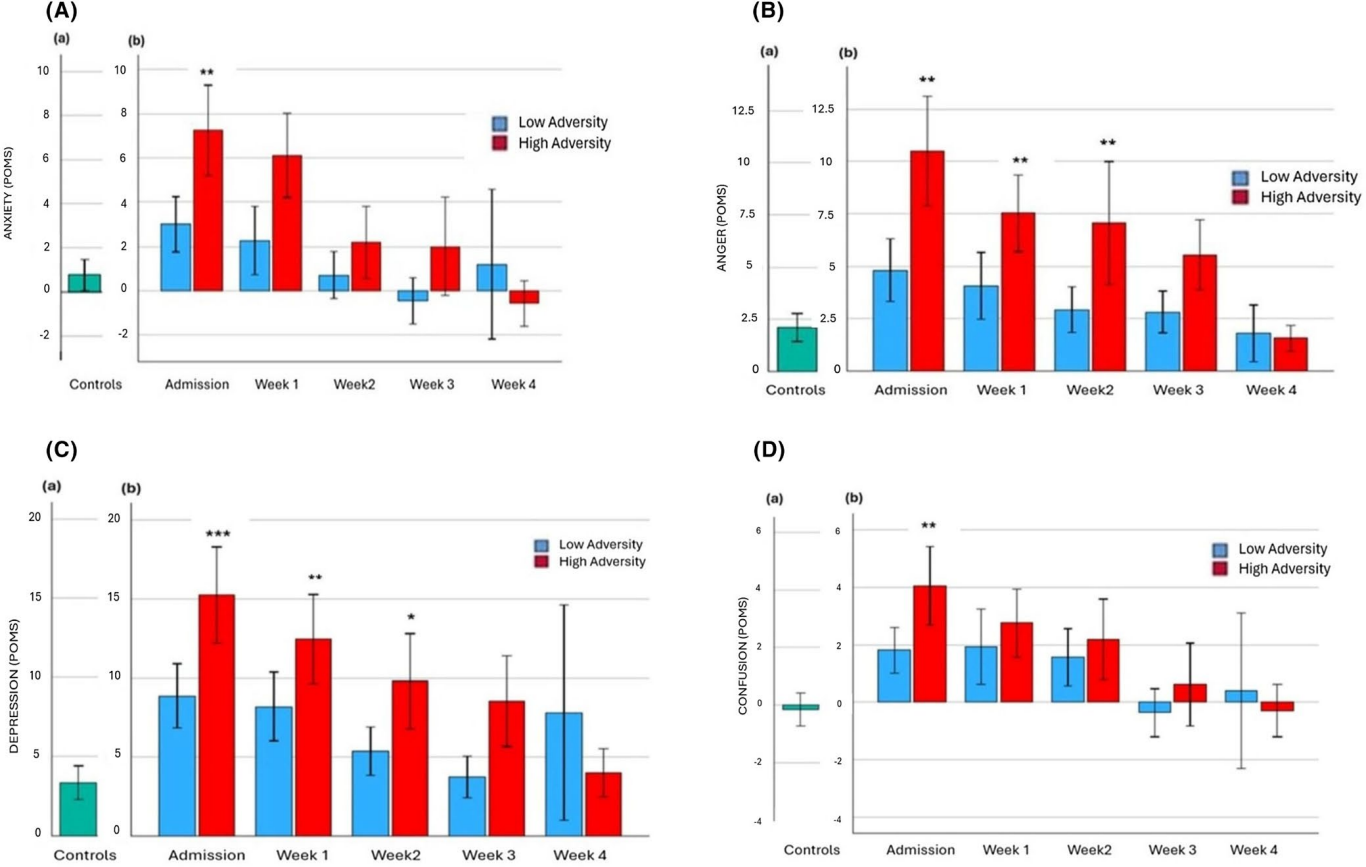
Profile of mood states (POMS). (a) Anxiety/Tension on admission to overnight stay in the control group (a) and across 4 weeks of inpatient stay in alcohol/adversity groups (b). A main effect of timepoint [*F* (4, 61) = 4.5, *p* = 0.006]. Admission >week 2 (*p* = 0.02) and week 3 (*p* = 0.009) and week 1 >week 3 (*p* = 0.02). ***p ≤ 0.001, **p ≤ 0.01, *p ≤ 0.05. All *p* values represent the difference between the alcohol groups and the control group. (b) Anger/Hostility on admission to overnight stay in the control group (a) and across 4 weeks of inpatient stay in alcohol/adversity groups (b). Interaction between continuous cumulative adversity scores × timepoint [*F* (4, 60) = 5.0, *p* < 0.001]. High adversity group >low adversity group on admission (*p* < 0.02). ***p ≤ 0.001, **p ≤ 0.01, *p ≤ 0.05. All *p* values represent the difference between the alcohol groups and the control group. (c) Depression/Dejection on admission to overnight stay in the control group (a) and across 4 weeks of inpatient stay in alcohol/adversity groups (b). A main effect of timepoint [*F* (4, 60) = 4.4, *p* = 0.006]. Admission >week 3 (*p* = 0.02). ***p ≤ 0.001, **p ≤ 0.01, *p ≤ 0.05. All *p* values represent the difference between the alcohol groups and the control group. (d) Confusion/Bewilderment on admission to overnight stay in the control group (a) and across 4 weeks of inpatient stay in alcohol/adversity groups (b). Interaction between continuous cumulative adversity scores × timepoint [*F* (1, 60) = 2.4, *p* = 0.005]. In the high adversity group, admission >week 3 (*p* < 0.04) and week 4 (*p* < 0.03). ***p ≤ 0.001, **p ≤ 0.01, *p ≤ 0.05. All *p* values represent the difference between the alcohol groups and the control group.

A main effect of Timepoint [*F* (4, 61) = 4.5, *p* = 0.006] showed that reported anxiety was higher on admission compared with Week 2 (*p* = 0.02) and Week 3 (*p* = 0.009) in the alcohol‐dependent individuals (Figure 3A). Anxiety was also higher in week 1 compared with week 3 (*p* = 0.02). No main effect of cumulative adversity or interaction with weekly timepoints was observed.

#### Analysis 2: Effects of Group (Controls vs. Low Adversity alcohol group vs. High Adversity alcohol group) on anxiety‐tension dimensional symptoms at all weekly timepoints

A main effect of Group on admission [*F* (2, 66) = 4.6, *p* = 0.01] showed that the High Adversity alcohol group reported significantly higher ratings of anxiety‐tension compared with the control group (*p* = 0.01). No significant effects of Group were observed at later timepoints.

### Anger‐hostility symptoms (POMS)

#### Analysis 1: Effects of cumulative lifetime adversity on anger‐hostility dimensional symptoms at all weekly timepoints in individuals with alcohol dependence

A main effect of Timepoint [*F* (4, 60) = 8.6, *p* < 0.001] indicated that the alcohol‐dependent participants reported significantly elevated levels of anger‐hostility on admission compared with week 1 (*p* < 0.03), week 2 (*p* < 0.02), week 3 (*p* < 0.001) and week 4 (*p* = 0.001) of inpatient stay (Figure 3B). A main effect of Cumulative Adversity (continuous score), [*F* (1, 60) = 2.7, *p* = 0.05] also indicated that as cumulative lifetime events increased, so did anger‐hostility across all timepoints. A significant interaction between Cumulative Lifetime Adversity (continuous score) × Timepoint interaction was also observed [*F* (4, 60) = 5.0, *p* < 0.001]. *Posthoc* analysis using High Adversity and Low Adversity groups indicated that the High Adversity alcohol group reported significantly higher anger‐hostility compared with the Low Adversity alcohol group on admission (*p* < 0.02). A trend only was observed in week 1 (*p* = 0.08) and week 2 (*p* = 0.09).

#### Analysis 2: Effects of Group (Controls vs. Low Adversity alcohol group vs. High Adversity alcohol group) on anger‐hostility dimensional symptoms at all weekly timepoints

A significant main effect of Group on admission [*F* (2, 67) = 12.3, *p* < 0.001] showed that the High Adversity alcohol group reported higher anger‐hostility compared with both the control group (*p* < 0.001) and the Low Adversity alcohol group (*p* = 0.01). The Low Adversity alcohol group also reported higher levels of anger‐hostility compared with the controls, but this was only a trend (*p* < 0.06). Main effects of Group were also observed at week 1 [*F* (2, 67) = 6.6, *p* = 0.003] and week 2 [*F* (2, 64) = 4.8, *p* = 0.01], where the High Adversity alcohol group again reported significantly higher anger‐hostility at both timepoints compared with the control group (*p* = 0.002 and *p* = 0.01, respectively).

### Depression‐dejection symptoms (POMS)

#### Analysis 1: Effects of cumulative lifetime adversity on depression‐dejection dimensional symptoms at all weekly timepoints in individuals with alcohol dependence

A main effect of Timepoint [*F* (4, 60) = 4.4, *p* = 0.006] indicated that greater depression‐dejection symptoms were reported on admission compared with week 3 of inpatient stay (*p* = 0.02) in alcohol‐dependent individuals (Figure 3C). No main effect of cumulative adversity or interaction with weekly timepoints was observed.

#### Analysis 2: Effects of Group (Controls vs. Low Adversity alcohol group vs. High Adversity alcohol group) on depression‐dejection dimensional symptoms at all weekly timepoints

A main effect of Group on admission [*F* (2, 67) = 8.1, *p* < 0.001], week 1 [*F* (2, 67) = 7.0, *p* = 0.002], and week 2 [*F* (2, 64) = 3.3, *p* < 0.05] showed that the High Adversity alcohol group reported higher depression‐dejection compared with the control group in all cases (*p* < .001; *p* = 0.001; *p* = 0.04, respectively).

### Fatigue‐inertia symptoms (POMS)

#### Analysis 1: Effects of cumulative lifetime adversity on fatigue‐inertia dimensional symptoms at all weekly timepoints in individuals with alcohol dependence

A significant interaction was observed between Cumulative Adverse Lifetime Events (CALE continuous score) and weekly timepoints [*F* (1, 60) = 2.2, *p* = 0.01]. *Posthoc* analysis using High Adversity and Low Adversity alcohol groups indicated that the High Adversity alcohol group reported significantly higher fatigue compared with the Low Adversity alcohol group on admission to inpatient treatment (*p* < 0.05). A trend only was observed in week 1 (*p* < 0.09).

#### Analysis 2: Effects of Group (Controls vs. Low Adversity alcohol group vs. High Adversity alcohol group) on fatigue‐inertia dimensional symptoms at all weekly timepoints

No significant main effect of Group was observed at any weekly timepoint.

### Confusion‐bewilderment symptoms (POMS)

#### Analysis 1: Effects of cumulative lifetime adversity on confusion‐bewilderment dimensional symptoms at all weekly timepoints in individuals with AD

A main effect of Timepoint [*F* (4, 60) = 4.4, *p* = 0.007] was observed where the alcohol‐dependent group reported higher confusion‐bewilderment on admission to inpatient stay compared with week 3 (*p* = 0.08, trend) (Figure 3D). A significant interaction between Cumulative Adverse Lifetime Events (CALE continuous score) and weekly timepoints was also observed [*F* (1, 60) = 2.4, *p* = 0.005]. *Posthoc* analysis using High Adversity and Low Adversity alcohol groups indicated that higher confusion symptoms were reported on admission compared with week 3 (*p* < 0.04) and week 4 (*p* < 0.03) of inpatient stay in the High Adversity alcohol group. These changes in confusion‐bewilderment scores across inpatient stay were not seen in the Low Adversity alcohol group. The High Adversity alcohol group also reported higher confusion‐bewilderment on admission compared with the Low Adversity alcohol group; however, this was only a trend (*p* = 0.06).

#### Analysis 2: Effects of Group (Controls vs. Low Adversity alcohol group vs. High Adversity alcohol group) on confusion‐bewilderment dimensional symptoms at all weekly timepoints

A main effect of Group on admission [*F* (2, 66) = 5.2, *p* < 0.008] was observed, which indicated that the High Adversity alcohol group reported significantly higher ratings of confusion‐bewilderment compared with the control group (*p* = 0.006).

### Vigor‐Activity Symptoms (POMS)

No significant effects were observed for the Vigor‐Activity subscale of the POMS.

### Alcohol withdrawal symptoms (CIWA)

#### Analysis 1: Effects of cumulative lifetime adversity on CIWA scores at all weekly timepoints in individuals with alcohol dependence

**FIGURE 4:**
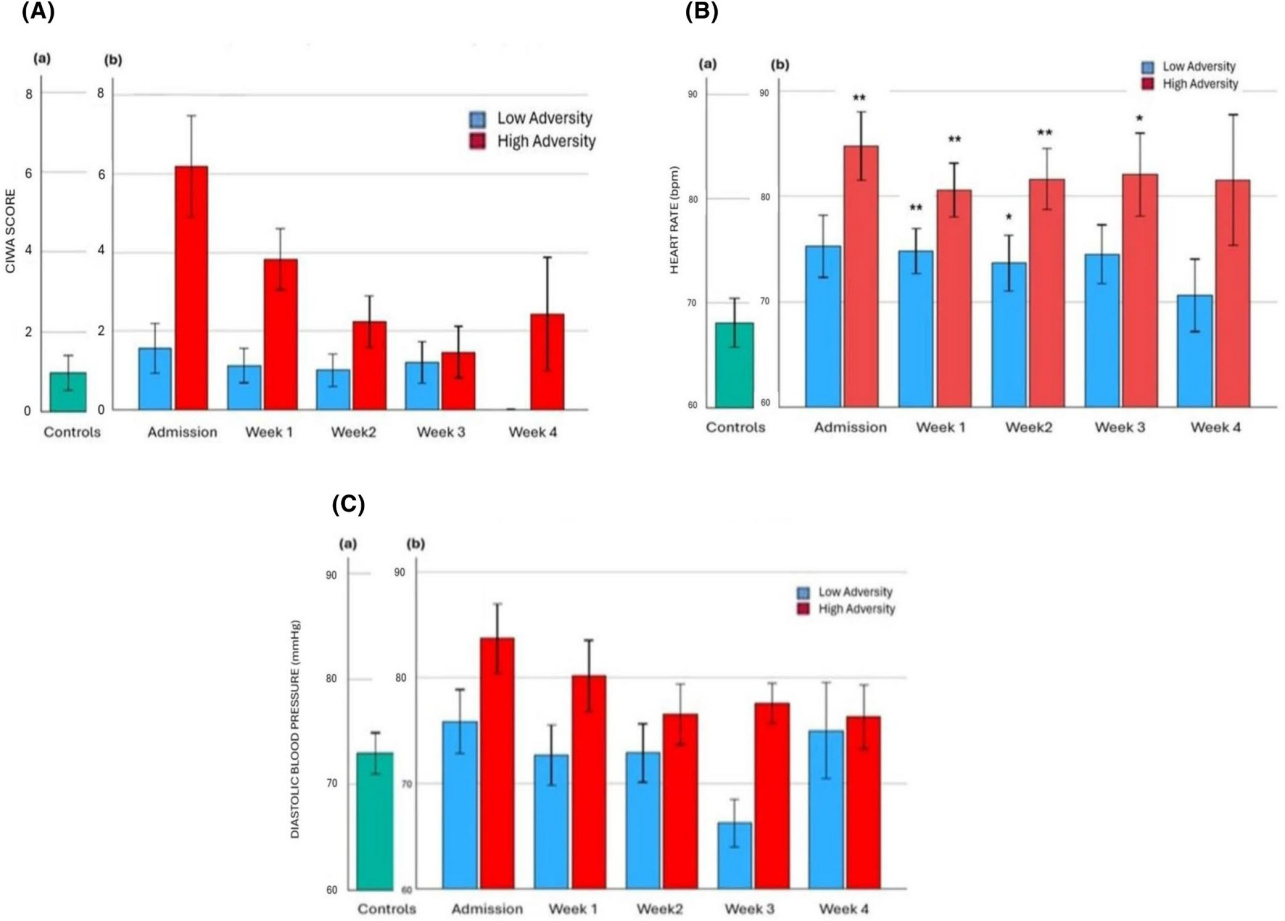
Clinical Institute Withdrawal Assessment (CIWA). (a) CIWA total score on admission to overnight stay in the control group (a) and across 4 weeks of inpatient stay in alcohol/adversity groups (b). Interaction between continuous cumulative adversity scores × timepoint [F (1, 65) = 1.9, *p* = 0.03]. High adversity group >low adversity group on admission (*p* < 0.001) and week 1 (*p* < 0.05). In the high adversity group, admission >week 1 (*p* < 0.03), week 2 (*p* < 0.001), week 3 (*p* < 0.001) and week 4 (*p* = 0.002). ***p ≤ 0.001, **p ≤ 0.01, *p ≤ 0.05. All *p* values represent the difference between the alcohol groups and the control group. (b) Heart rate on admission to overnight stay in the control group (a) and across 4 weeks of inpatient stay in alcohol/adversity groups (b). ***p ≤ 0.001, **p ≤ 0.01, *p ≤ 0.05. All *p* values represent the difference between the alcohol groups and the control group. (c) Diastolic blood pressure on admission to overnight stay in the control group (a) and across 4 weeks of inpatient stay in alcohol/adversity groups (b). ***p ≤ 0.001, **p ≤ 0.01, *p ≤ 0.05. All *p* values represent the difference between the alcohol groups and the control group.

A main effect of Timepoint [*F* (4, 65) = 13.4, *p* < 0.001] indicated that withdrawal symptoms on admission were significantly higher in the alcohol‐dependent participants compared with all other inpatient weeks (*p* < 0.03; *p* < 0.001; *p* < 0.001; *p* = 0.01, respectively) (Figure 4A). A significant Cumulative Adverse Lifetime Events (continuous score) × timepoint interaction [*F* (1, 65) = 1.9, *p* = 0.03] was also observed. *Posthoc* analysis using High Adversity and Low Adversity alcohol groups indicated that the High Adversity alcohol group reported significantly higher withdrawal symptoms on admission (*p* < 0.001) and at week 1 (*p* < 0.05) of inpatient stay compared with the Low Adversity alcohol group. Additionally, higher withdrawal symptoms were observed on admission compared with all other inpatient weeks in the High Adversity alcohol group only (*p* < 0.03; *p* < 0.001; *p* < 0.001; *p* = 0.002). No differences in symptoms were seen across timepoints in the Low Adversity alcohol group.

#### Analysis 2: Effects of Group (Controls vs. Low Adversity alcohol group vs. High Adversity alcohol group) on CIWA scores at all weekly timepoints

A main effect of Group on admission [*F* (1, 65) = 1.9, *p* = 0.03], indicated that the High Adversity alcohol group reported higher CIWA symptoms compared with the Low Adversity alcohol group (*p* = 0.03).

### Heart Rate (CIWA)

#### Analysis 1: Effects of cumulative lifetime adversity on heart rate scores at all weekly timepoints in individuals with alcohol dependence

No main effect of cumulative adversity or interaction with weekly timepoints was observed (Figure 4B).

#### Analysis 2: Effects of Group (Controls vs. Low Adversity alcohol group vs. High Adversity alcohol group) on heart rate at all weekly timepoints

A main effect of Group was observed on admission, [*F* (2, 52) = 4.9, *p* = 0.01] week 1[*F* (2, 53) = 8.5, *p* < 0.001], week 2 [*F* (2, 53) = 7.5, *p* = 0.001], and week 3 [*F* (2, 43) = 4.7, *p* < 0.02]. In all cases, the High Adversity alcohol group demonstrated significantly elevated beats per minute (bpm) compared with the control group (*p* < 0.05, in all cases). The Low Adversity alcohol group also showed elevated bpm compared with the control group in week 1 and week 2 (*p* < 0.05, in all cases).

### Systolic blood pressure (SBP**)**

#### Analysis 1: Effects of cumulative lifetime adversity on SBP at all weekly timepoints in individuals with alcohol dependence

A significant main effect of Timepoint was observed [*F* (4, 25.2) = 3.4, *p* = 0.02]; however, following Bonferroni correction, no significant differences in SBP across timepoints were observed.

#### Analysis 2: Effects of Group (Controls vs. Low Adversity alcohol group vs. High Adversity alcohol group) on SBP at all weekly timepoints

No main effect of Group was observed.

### Diastolic blood pressure (DBP)

#### Analysis 1: Effects of cumulative lifetime adversity on DBP at all weekly timepoints in individuals with alcohol dependence

No main effect of cumulative adversity or interaction with weekly timepoints was observed (Figure 4C).

#### Analysis 2: Effects of Group (Controls vs. Low Adversity alcohol group vs. High Adversity alcohol group) on DBP at all weekly timepoints

A main effect of Group was observed at Week 1 [*F* (2, 53) = 3.2, *p* = 0.05] and Week 3 [*F* (2, 43) = 4.3, *p* = 0.02], where the High Adversity alcohol group demonstrated higher DBP compared with the Low Adversity alcohol group in both cases (*p* = 0.05 and p < 0.02, respectively).

## DISCUSSION

This study investigated the pattern of early alcohol abstinence symptoms in adult inpatients with alcohol dependence, focusing on alcohol craving, mood, withdrawal symptoms, including cardiovascular measures over 3 to 4 weeks. A central aim was to explore how cumulative life adversity might moderate these symptoms, with the expectation that individuals experiencing a greater number of lifetime adverse events would exhibit greater symptom severity. Results demonstrated that alcohol‐dependent patients showed significant improvements in alcohol cravings, depression, anxiety, and confusion scores by week three of treatment, consistent with the timeframe reported in previous research findings (Heilig et al., 2010; Schuckit & Hesselbrock, 1994; Wetterling & Junghanns, 2000). Exceptions included self‐reported fatigue, anger, and withdrawal scores which decreased within the initial week of treatment. Participants with higher cumulative adversity also exhibited a significantly more severe pattern of abstinence symptoms in relation to all measures compared with controls. With the exception of alcohol craving and heart rate, this discrepancy was not observed between alcohol‐dependent individuals experiencing fewer adverse events and controls.

Alcohol‐dependent individuals with greater lifetime adversity also reported elevated anger‐hostility, fatigue‐inertia, confusion‐bewilderment, and CIWA withdrawal symptoms relative to those with fewer lifetime adverse events on admission to inpatient treatment. Importantly, cumulative adversity scores impacted these symptoms on admission even after controlling for recent alcohol and tobacco consumption over the preceding 3 months, suggesting that cumulative adversity may play a role in determining individual responses to alcohol abstinence initiation and possibly serve as a prognostic marker for relapse‐related clinical symptoms during early recovery.

While clinical symptoms were significantly heightened in the high adversity group compared with controls, many elevated symptoms were remarkably brief. Specifically, anger‐hostility, fatigue‐inertia, confusion‐bewilderment, and withdrawal symptoms resolved to control levels within just a few days. Despite their transient nature, however, these elevated symptoms may still represent clinically important changes. For example, evidence‐based research demonstrates that elevated levels of alcohol abstinence symptoms and craving at treatment entry are associated with heavy drinking patterns and poor clinical outcomes during treatment (Martins et al., 2022), and can also influence medication effectiveness (Anton et al., 2020; Sinha et al., 2021). Additionally, elevated depressive symptoms on admission to treatment have been associated with various negative treatment outcomes, regardless of subsequent symptomatic improvement during the course of treatment (Fox et al., 2020).

During the third week of inpatient treatment, decreased alcohol craving was observed exclusively among alcohol‐dependent patients who had higher levels of cumulative lifetime adversity. This is due to the fact that these individuals initially reported more intense cravings both at treatment admission and during the first week compared to those with lower adversity exposure and control participants, respectively. In contrast, patients with less lifetime adversity showed neither elevated initial craving levels nor subsequent reductions. This pattern has important clinical implications, as alcohol craving represents both a hallmark symptom of early withdrawal and a robust predictor of treatment dropout and relapse risk (Moore et al., 2014; Panlilio et al., 2019; Sinha et al., 2006). In addition, research has demonstrated that elevated craving levels during the first 6 weeks of treatment as well as on admission can influence how patients respond to standard, FDA‐approved, alcohol pharmacotherapy such as naltrexone (Monterosso et al., 2001; Richardson et al., 2008).

Recent work by Martins et al. (2022) also highlights the clinical importance of elevations in craving during early abstinence. For example, their investigation revealed that higher pretreatment craving levels predicted both increased heavy drinking episodes and accelerated return to problematic drinking during an 8‐week relapse prevention program, even after controlling for baseline drinking severity. The study also identified an interaction between withdrawal symptoms and treatment response, such that participants with lower CIWA scores at treatment entry showed greater drinking reductions throughout the trial compared to those with more severe withdrawal symptoms. Similarly, research has indicated that early withdrawal symptom severity can influence treatment response to prazosin in individuals with alcohol use disorder (Sinha et al., 2021). These convergent findings suggest that the constellation of very early abstinence symptoms such as craving intensity and mood disturbances in alcohol‐dependent individuals with a high number of lifetime adverse events may serve as meaningful predictors of treatment trajectory and outcomes. Consequently, determining cumulative adversity could offer clinicians a practical tool for anticipating the heterogeneity of early abstinence presentations and, by extension, inform treatment prognosis and planning.

Regarding the physiological metrics of early alcohol abstinence, alcohol‐dependent individuals with higher cumulative adversity demonstrated a slightly more elevated and persistent heart rate pattern compared with the low adversity group. While the high cumulative adversity alcohol group demonstrated elevated bpm on admission to treatment and during weeks 1, 2, and 3 compared with controls, the low adversity group only demonstrated elevated bpm compared with controls at weeks 1 and 2. Again, analyses controlled for severity of alcohol and tobacco use in the 3 months prior to treatment admission. In terms of clinical implications, faster beats per minute in both alcohol‐dependent groups may reflect several key clinical features, including low heart rate variability (Tiwari et al., 2021) and dampened phasic response to stress during early abstinence (Croissant et al., 2006; Sinha et al., 2009), both of which have been linked to relapse vulnerability and poor overall health outcomes in a range of problem drinking populations (Frasier et al., 2024; Ralevski et al., 2019). In terms of blood pressure measurements, alcohol‐dependent individuals with high adversity also demonstrated elevated diastolic blood pressure (DBP) during the first and third weeks of treatment compared to those with lower adversity scores.

Despite heart rate and DBP differences reaching statistical significance, it is important to note that all cardiovascular measurements remained within normotensive ranges across both high and low adversity groups. This raises important questions about the clinical relevance of these subtle variations and highlights the need for additional research to fully determine whether such cardiovascular changes represent meaningful subclinical adaptations. Support for such subtle, but salient adaptations, is provided by studies which have demonstrated that tonic and phasic stress and autonomic system dysregulation within normal parameters can still predict craving intensity and relapse likelihood (Chen et al., 2020; Fox et al., 2007; Sinha et al., 2009). Findings such as these may help support the idea that cumulative adversity may also reflect subtle physiological adaptations that serve as broad indicators of treatment response and recovery trajectory.

In the alcohol‐dependent inpatients, systolic blood pressure (SBP) showed a modest decline during weeks 2 and 3 relative to admission values, which is a pattern that aligns with prior research demonstrating blood pressure reductions following alcohol cessation within the first month of abstinence (Aguilera M. et al., 1999; Stewart et al., 2008). However, these changes did not maintain statistical significance after applying Bonferroni correction for multiple comparisons. The absence of significant SBP changes may be attributed to sex‐related confounding effects identified in the statistical model, where men had significantly higher SBP compared with women. Since men comprised most participants in the alcohol group, but not the control group, this may have masked the anticipated decrease in blood pressure improvements.

This study has several important limitations. The small sample and limited representation of women in the alcohol‐dependent groups means our findings are constrained regarding generalizability. Future research should additionally examine which types of cumulative adversity most strongly influence withdrawal symptoms. While counting adverse events offers a straightforward, quantifiable measure suitable for clinical screening, some researchers suggest that the “dosing” or intensity of adversity may be more critical than simple “accumulation” in determining mental health outcomes (Künzi et al., 2022). The present study did not directly measure the intensity and specific nature of adverse experiences; however, participants in the high adversity group demonstrated significantly elevated scores across all CALE subscales relative to the low adversity group, indicating broad‐based increases in adverse experiences rather than elevated scores in any single domain. Notably, however, the most striking difference between the adversity groups emerged in life trauma exposure, with the High Adversity participants reporting an average of 15.2 traumatic events compared to 6.1 in controls. This substantial disparity suggests that traumatic stress may have uniquely disruptive effects on recovery processes, underscoring the potential importance of identifying and addressing trauma during early recovery phases. Although this research was focused on quantifying adverse events, future investigations that examine the more complex relationships between different types of adversity and their varying levels of severity are certainly encouraged.

Limitations notwithstanding, this study provides valuable insights by documenting early alcohol abstinence patterns over 3 to 4 weeks of complete sobriety, with socially drinking controls maintained in comparable overnight conditions. This approach is particularly noteworthy because early abstinence symptoms serve as important predictors of treatment outcomes, yet they are neither well characterized nor systematically monitored in typical inpatient or outpatient treatment settings. The study also demonstrates the potential clinical utility of cumulative lifetime adversity as a readily assessable, objective factor that may predict the severity of craving, mood disturbances, and subclinical cardiovascular risk indicators during withdrawal. Identifying such markers of early withdrawal variability represents a crucial step toward developing more individualized and effective treatments for alcohol use disorders. Continued research focused on identifying factors that contribute to withdrawal heterogeneity will be essential for advancing personalized treatment approaches and improving outcomes for individuals with alcohol use disorder.

## FUNDING INFORMATION

This study was supported in parts by grants: R01AA20095 (Fox); R21AA024880 (Fox); R03AA022500 (Fox); HRSA D40HP33382 (Hyman); R01AA20504 (Sinha).

## CONFLICT OF INTEREST STATEMENT

The authors declare no conflict of interest with respect to thier authorship or publication of this article.

## Data Availability

The data that support the findings of this study are available from the corresponding author upon reasonable request.
